# What Older People Like to Play: Genre Preferences and Acceptance of Casual Games

**DOI:** 10.2196/games.7025

**Published:** 2017-04-18

**Authors:** Alvin Chesham, Patric Wyss, René Martin Müri, Urs Peter Mosimann, Tobias Nef

**Affiliations:** ^1^ Gerontechnology & Rehabilitation University of Bern Bern Switzerland; ^2^ University Hospital of Old Age Psychiatry University of Bern Bern Switzerland; ^3^ Perception and Eye Movement Laboratory Division of Cognitive and Restorative Neurology, Department of Neurology University Hospital Inselspital Bern Switzerland; ^4^ ARTORG Center for Biomedical Engineering Research University of Bern Bern Switzerland

**Keywords:** games, recreational, genre preference, mobile applications, aged, core elements of the gaming experience

## Abstract

**Background:**

In recent computerized cognitive training studies, video games have emerged as a promising tool that can benefit cognitive function and well-being. Whereas most video game training studies have used first-person shooter (FPS) action video games, subsequent studies found that older adults dislike this type of game and generally prefer casual video games (CVGs), which are a subtype of video games that are easy to learn and use simple rules and interfaces. Like other video games, CVGs are organized into genres (eg, puzzle games) based on the rule-directed interaction with the game. Importantly, game genre not only influences the ease of interaction and cognitive abilities CVGs demand, but also affects whether older adults are willing to play any particular genre. To date, studies looking at how different CVG genres resonate with older adults are lacking.

**Objective:**

The aim of this study was to investigate how much older adults enjoy different CVG genres and how favorably their CVG characteristics are rated.

**Methods:**

A total of 16 healthy adults aged 65 years and above playtested 7 CVGs from 4 genres: casual action, puzzle, simulation, and strategy video games. Thereafter, they rated casual game preference and acceptance of casual game characteristics using 4 scales from the Core Elements of the Gaming Experience Questionnaire (CEGEQ). For this, participants rated how much they liked the game (enjoyment), understood the rules of the game (game-play), learned to manipulate the game (control), and make the game their own (ownership).

**Results:**

Overall, enjoyment and acceptance of casual game characteristics was high and significantly above the midpoint of the rating scale for all CVG genres. Mixed model analyses revealed that ratings of enjoyment and casual game characteristics were significantly influenced by CVG genre. Participants’ mean enjoyment of casual puzzle games (mean 0.95 out of 1.00) was significantly higher than that for casual simulation games (mean 0.75 and 0.73). For casual game characteristics, casual puzzle and simulation games were given significantly higher game-play ratings than casual action games. Similarly, participants’ control ratings for casual puzzle games were significantly higher than that for casual action and simulation games. Finally, ownership was rated significantly higher for casual puzzle and strategy games than for casual action games.

**Conclusions:**

The findings of this study show that CVGs have characteristics that are suitable and enjoyable for older adults. In addition, genre was found to influence enjoyment and ratings of CVG characteristics, indicating that puzzle games are particularly easy to understand, learn, and play, and are enjoyable. Future studies should continue exploring the potential of CVG interventions for older adults in improving cognitive function, everyday functioning, and well-being. We see particular potential for CVGs in people suffering from cognitive impairment due to dementia or brain injury.

## Introduction

### Video Game Training

Commercial video games are designed to be enjoyable, challenging, and capable of fostering sustained player engagement [1]. Video games are further subdivided into “hardcore” and “casual” video games (CVGs). Hardcore video games are complex, require high commitment, and are played for longer periods of time, whereas CVGs are simple, require low commitment, and have short play sessions [2]. Traditionally, video games are not designed for specific improvement of cognitive domains. An exception to this are cognitive exercises, or brain-trainers, that use gamification of cognitive training, where neuropsychological tests are combined with video games elements such as scorekeeping and leaderboards [3]. Cognitive benefits from playing video games can therefore be considered more of an unintentional by-product [1].

To date, most video game training studies have used hardcore action video games (especially first-person shooter [FPS]) to demonstrate how video games can improve perceptual and cognitive abilities, notably those that are also subject to age-associated decline [4,5]. Although action video games are mainly targeted at younger audiences, these kinds of games present barriers to older adults. First, learning to play and interact with fast-paced action video games can be very difficult and demotivating for older adults. Second, questionnaire and playtest studies have found that older adults generally dislike action video games, especially when featuring violent content [4,6-8]. Together, this leads to a situation where older adults, despite promising cognitive benefits, are less willing and motivated to play action video games, and thus less likely to follow through with action video game interventions [4,8,9].

In order to offer older adults and even patients more attractive forms of video game interventions, we suggest tapping the potential of CVGs as an enjoyable activity to improve cognitive functions and emotional well-being. By better understanding the types of CVGs older adults enjoy playing and the specific game characteristics they find appealing, we hope to identify CVG genres and game characteristics that might raise the motivation of participants in future video game interventions.

### The Case for Causal Video Games

What makes CVGs an excellent choice for an older adult population is their promise to “eliminate any possible barrier to someone enjoying the game” [10]. Unlike hardcore video games, CVGs are intended as games for everyone that are easy to use and play, do not require high commitment or special skills, and can be completed in short play sessions [11,12].

To reach this goal, CVGs follow four casual game design values: “Acceptability” refers to the appeal for a wider, heterogeneous group of players. To this end, CVGs borrow themes familiar to the social context of the player that are nonviolent and foster positive emotions and growth. “Accessibility” makes sure that players with different cognitive and physical skill levels can quickly learn to play the game. “Simplicity” aims to lower the cognitive load on the player through simple and minimized game elements as well as easy rules and goals. Finally, “flexibility” assures that CVGs adapt to players and integrate into their everyday life. For this, CVGs are designed to be error-forgiving, adapt the difficulty level to the player, and can be easily stopped and replayed [11,13,14].

Interestingly, CVGs further try to provide players with positive outcomes outside of the game such as mental exercise, relaxation, social and playful activity [11]. This touches a recently published “gerontoludic” manifesto [9] that suggests focusing more on whether video games create an enjoyable experience and consider the preferences of older adults (ie, the “playfulness” aspect) rather than pragmatically insisting on improvement of cognitive abilities (ie, the “usefulness” aspect) [9] and age-related barriers to interact with video games (ie, the “accessibility” aspect). Given that recent studies suggest that CVGs have a potential in improving cognitive function and promoting emotional well-being [3], this study aims to look at whether different CVG genres are suitable and fun to play for older adults.

### Casual Video Game Genres

The notion of video game genre that allows organizing games into categories is crucial in connection with CVGs for three reasons: Whereas it is agreed that CVGs, as a whole, reduce usability barriers and are the most enjoyed and motivating type of video games, research into preferences for CVG subgenres among older adults is lacking [15]. This is reflected even among active older gamers that were shown to predominantly play card or board game-like video games, whereas other genres are rarely played [16]. Second, recent studies have shown that different CVG genres engage different perceptual and cognitive functions, allowing the selection of specific game genres to improve specific cognitive skills [17-19]. From a pragmatic point of view, this is crucial as video game-based interventions will not benefit cognitive abilities unless the game is known to engage specific cognitive skills [4]. By better understanding game genre preferences of older adults, future game interventions could offer them different CVG genres that they are both willing to play and that improve specific cognitive abilities [4,20]. Third, the concept of genre closely relates to usability. As the pattern of interactions and rules of the games represent the most commonly used video game genre classification scheme [21], examining how older adults learn, control, and understand games from different genres can help addressing usability problems pertaining to each video game genre [22].

Although several recent studies have addressed usability barriers and used survey methods to assess game preferences in older adults [4,16,23,24], playtest studies using a wider range of CVG genres to study game interests and preferences of older adults are lacking [4].

### Research Questions

In this study, we wanted to identify CVG genres that older adults enjoy and are willing to play most, and how favorably their CVG design values are rated. Given the explorative nature of this study, the following research questions rather than hypotheses were formulated. Do the CVGs provide enjoyment and is there a preference for specific CVG genres? Do the CVGs meet the casual game design values (easy rules and story, clear goals and actions, easy to control, and make their own) and are they suitable for older adults?

## Methods

### Participants

In total, 16 healthy older adults (5 females, 11 males) aged between 65 and 84 years (mean 71.94 years, SD 6.34) participated in this study. Participants were recruited from the Seniors University of Bern, Switzerland. All participants had normal or corrected-to-normal vision. Exclusion criteria were a diagnosis of dementia or mild cognitive impairment and fine motor skill impairments leading to inability to handle a tablet computer. All participants provided signed informed consent in accordance with the latest version of the Declaration of Helsinki and were rewarded with two cinema tickets for their participation. The cantonal ethics committee of Bern, Switzerland (Kantonale Ethikkommision) granted the ethics approval.

### Selected Casual Video Games

**Figure 1:**
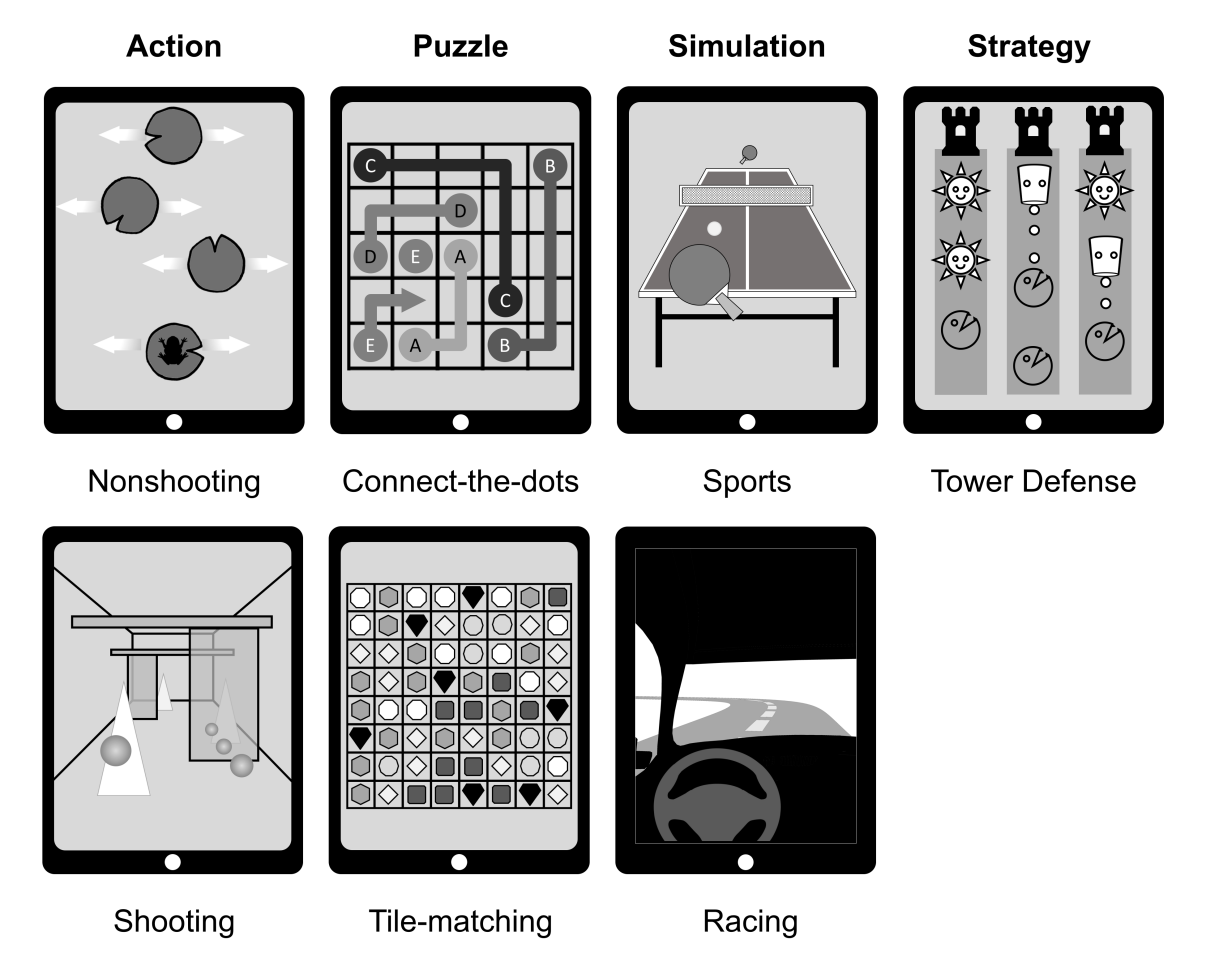
Casual video game genres (illustrations based on video games used in the study).

For this study, we selected 7 single-player, tablet-based CVGs (Figure 1) available on the Web on app stores for mobile phones and tablet computers (iTunes App store, Apple Inc). The CVGs were selected by a professional game designer to conform to accepted casual game design values [11,13]. These include familiar, cheerful, and nonviolent game topics (“acceptability”), games that are simple to play and learn with easy rules and goals (“simplicity”), allow players to quickly reach proficiency (“accessibility”), and provide a flexible and error-forgiving game experience (“flexibility”).

For genre-specific comparisons, we selected casual games from 4 casual game genres (casual action, casual puzzle, casual simulation, and casual strategy games), following video game genre classifications based on both the cognitive skills they involve [25] and the pattern of interactions and rules of the games [21]. Casual action games require the player to perform a series of actions to meet specific objectives and usually involve eye-hand coordination and fast reaction [25,26]. Casual puzzle games refer to games with the goal to solve enigmas via manipulation of game objects and require reasoning and problem-solving skills [21,25]. Casual simulation games recreate real-world activities (eg, sports, driving, and city building) and require domain-specific and procedural knowledge about the system, coordination of cognition, information processing, as well as movement control [19,21,25,27]. Finally, casual strategy games require planning, decision making, and execution and adjustments of actions to achieve a desired outcome in the system and require executive control [25].

For the casual action games (Figure 1, first row from left), we chose an FPS and a nonshooting game. In the no shooting action game Pocket Frog Splash Sliders (Nimblebit), players must hop a frog across lily pads that move back and forth by tapping the touchscreen in time. Missing the lily pad will subtract 1 life from the player’s lives, whereas extra lives could be earned by skipping lily pads during a jump. The shooting action game Smash Hit (Mediocre AB), played in training mode, combines an infinite runner and FPS game elements. The goal is to move ahead as far as possible by collecting bullets and hitting glass obstacles by tapping the touchscreen to aim and shoot.

For the puzzle genre (Figure 1, second row from left), we chose 2 grid-based puzzles differing in interaction with the game. Flow Free (Big Duck Games LLC) is a logic puzzle game in which players must connect pairs of same-colored dots, using tap and drag movements. To solve the puzzle, the entire grid should be filled with nonoverlapping connections to cover the entire grid using a minimal number of moves [28,29]. Bejeweled (PopCap Games), played in infinite mode, is a tile-matching puzzle game played on an 8×8 grid covered with jewels in 7 different colors and shapes. The player must swap two adjacent gems (ie, 1 of the 4 cardinal neighbors) to create an alignment (vertical or horizontal) of 3 or more identical gems. Matched gems are removed and newly generated gems fall in their place [30].

For the casual simulation (Figure 1, third row from left), we chose a racing and sports simulation game. Real Racing 3 (Firemonkeys Studios) is a realistic racing game with different racing events. The goal of the game is to win the race and steer the car by tilting the tablet left to turn left and right to turn right. The car accelerates and decelerates automatically. For this study, we selected a beginner-level race (Circuit de Spa-Francorchamps). Virtual Table Tennis HD (SenseDevil Games) is a realistic sports simulation game that emulates table tennis in the game world. The game is played against a computer opponent by moving the ping-pong paddle along the touchscreen using drag movements. For this study, we selected a game from the beginner level.

For the casual strategy game genre (Figure 1, fourth row from left), we selected the Plants vs Zombies (PopCap Games) tower defense (TD) game, a subgenre of real-time strategy games. Due to time constraints and restraint from military-themes and games that require prolonged commitment, we chose one casual strategy game only. The goal of TD games is to collect resources (suns) and place defensive units (plants) along paths on a map to prevent enemies (zombies) attacking on parallel lanes from reaching the player’s base. The game is lost when the enemy reaches the player’s home base; the game is won when waves of attacking enemies are successfully defended [31].

### Questionnaire

The players’ casual game experience was measured using the Core Elements of the Gaming Experience Questionnaire (CEGEQ) after completion of each game [32]. The CEGEQ is based on the assumption that enjoyment of a game emerges from the player’s perception of the video game and their interactions with it. Here, the player’s perception of the game (ie, video game) is assumed to be formed by the environment (eg, graphics and sounds) and game-play (eg, rules and scenario) that are both thought to produce enjoyment. The player’s interaction with the game (“puppetry”) is believed to reflect ownership, which is achieved through manipulation of the game (“control”) or can be produced by “facilitators” that can compensate lacking control over the game [32]. The CEGE questionnaire contains 10 scales representing the latent constructs (enjoyment, frustration, CEGE, puppetry, video game, control, facilitators, ownership, game-play, and environment) measured with 38 items. The reliability of the CEGEQ is sufficiently high (Cronbach alpha=.794) [33].

For this study, we selected 5 scales of the CEGE (enjoyment, game-play, control, ownership, and environment). Casual game genre preference was measured using the enjoyment scale (3 items) reflecting the extent to which each game was enjoyed and encouraged replayability. Three scales were used to examine to what extent the selected casual games met the casual game values [11,13,34] as rated by the participants. The game-play scale (6 items) reflects how the rules and underlying story or scenario of the game are judged. Control (8 items) refers to how the player learns to control the game and make it his own, and measures whether the general goal and actions of the game are clear, how easy the controllers (input device) are to use, how they are mapped to the actions, and whether everything was visible on the interface. The scale ownership (6 items) was used to determine how the player uses the actions to complete the goal of the game, creates a strategy and personal goals, uses rewards, and takes ownership over the game. Finally, environment (6 items), the way the player perceives the game via graphics and sounds, was measured. This was done to rule out effects attributable artistic style and visual aesthetics that are inherent to video game genre, but not of interest to our study.

### Procedure

This study used playtesting sessions that combine questionnaires with the opportunity of hands-on playing of video games to quantify the player’s attitudes, opinions, and perception of different CVG genres. This approach is well suited for persons with no previous game experience and also far more informative than mere interviews and surveys in active gamers [35]. For the informal playtesting session (lasting about 120 min), participants were tested individually and were seated at a desk next to the experimenter in a small laboratory room. All casual games and video clips were administered on a tablet computer with touch interface and a 9.7 screen (iPad Air 2, Apple Inc). For the playtesting session, participants were first asked to fill out questionnaires regarding demographics, game, and computer experience. After that, participants were informed about the procedures of the playtesting study and a written consent was obtained. For each of the 7 CVGs, participants first watched a short video clip of a person playing the game (observational session, 3-5 min). After that, the experimenter read the instructions to the participant to ensure that they understood the rules. Then, the participants were invited to play the game for a limited amount of time or levels (game-play session, about 5 min). Following that, participants were asked to evaluate their game experience with the CVG by answering the 39 items of the CEGEQ on a closed 5-point Likert-type scale, ranging from 1 (completely disagree) to 5 (completely agree), which lasted between 5 and 10 min.

### Statistical Analysis

The participant’s evaluation of the game experience with the casual games was assessed with 39 closed-response questions that participants answered using a closed 5-point Likert-type scale. The 10 negatively worded items were reverse-coded by subtracting the value from 6. Scores for each of the 10 scales of the CEGEQ were then calculated by averaging the item ratings corresponding to each scale and dividing them by 5. We removed item 24 (“I felt guilty for the actions in the game”) from the CEGEQ as we specifically selected nonviolent video games.

First, separate repeated measures one-way Analyses of Variance (rmANOVA) with casual game genre as a within-subject factor were performed by using SPSS for Windows (version 24.0, SPSS Inc) to test whether the video game genre had a significant effect on each of the 5 CEGEQ scales of interest (enjoyment, game-play, control, ownership, and environment). An alpha value of .05 was used to determine significance.

Second, linear mixed effect models (LMEM), fit by restricted maximum likelihood estimation (REML) were performed (using R version 3.3.1 (R Foundation for Statistical Computing, Vienna, Austria ) and the package lme4 [36]) for each CEGE scale (enjoyment, game-play, control, and ownership) to examine the effects of environment ratings, game experience, age, and level of education on CVG genre. To this purpose, we fitted an LMEM with the respective CEGE scale as dependent variable, a random intercept per subject as random effect, and video game genre, environment ratings, game experience, age, and education as fixed effects. Gender was not included as a fixed parameter due to the small number of female participants. The global effect of the factor video game genre was tested using the conditional *F* test, whereas all other fixed-effect parameters were tested using a conditional *t* test. Pairwise comparisons of the estimated marginal means were performed between the different video game genres using Bonferroni-Holm correction for multiple testing.

## Results

### Demographics

All 16 participants reported having access to and using desktop or laptop computers on a daily or weekly basis, whereas only 5 (5/16, 27.8%) participants (2 females, 3 males) reported having ever played, and that they are currently playing computer video games. All 5 participants reported playing games preinstalled on Desktop PC computers (eg, Free Cell, Patience, and Mahjong) on a daily to monthly basis. None of the participants reported having ever played video games on tablet-computers.

### Ranking of the Casual Games on the Enjoyment Scale

The results of this study indicate that the mean participant’s ratings of the casual games were above average (ie, >0.5—the midpoint of the item rating range) across all CEGE scales considered in the analysis. The order of casual game enjoyment or genre preference from the most enjoyed to the least enjoyed was as follows (Table 1, total): casual puzzle games (Flow Free followed by Bejeweled); the casual action no shooting game (Pocket Frog Splash); the casual action shooting (Smash Hit); and casual strategy game (Plants vs Zombies) that showed identical ratings, and, finally, the casual simulation games (Virtual Table Tennis followed by Real Racing).

**Table 1:** Means and CIs (95%) of the selected CEGE scales for all casual games played grouped by gender (females: n=5; males: n=11; and total: N=16).

Genre	Gender	Enjoyment	Game-play	Control	Ownership	Environment
**Casual action video games**						
No shooting	Total	0.85 (0.79-0.92)	0.61 (0.53-0.69)	0.72 (0.67-0.78)	0.64 (0.58-0.70)	0.72 (0.66-0.78)
	Female	0.87 (0.77-0.96)	0.55 (0.45-0.64)	0.71 (0.68-0.74)	0.56 (0.52-0.59)	0.73 (0.67-0.79)
	Male	0.85 (0.78-0.92)	0.64 (0.56-0.74)	0.73 (0.65-0.79)	0.68 (0.62-0.74)	0.72 (0.65-0.79)
Shooting	Total	0.83 (0.71-0.94)	0.66 (0.59-0.73)	0.72 (0.68-0.77)	0.70 (0.63-0.77)	0.76 (0.71-0.81)
	Female	0.79 (0.49-1.00)	0.57 (0.51-0.63)	0.70 (0.64-0.78)	0.70 (0.63-0.82)	0.75 (0.68-0.81)
	Male	0.85 (0.75-0.93)	0.70 (0.62-0.78)	0.74 (0.69-0.79)	0.69 (0.62-0.77)	0.76 (0.70-0.83)
**Casual puzzle video games**						
Connect-the-dots	Total	0.95 (0.91-0.99)	0.65 (0.60-0.71)	0.85 (0.80-0.90)	0.77 (0.71-0.83)	0.78 (0.73-0.84)
	Female	0.93 (0.85-1.00)	0.64 (0.55-0.73)	0.84 (0.76-0.92)	0.78 (0.73-0.83)	0.75 (0.68-0.81)
	Male	0.95 (0.91-0.99)	0.66 (0.61-0.72)	0.86 (0.8-0.92)	0.76 (0.68-0.84)	0.80 (0.74-0.86)
Tile-match	Total	0.85 (0.78-0.92)	0.70 (0.62-0.78)	0.79 (0.74-0.84)	0.70 (0.65-0.75)	0.75 (0.68-0.81)
	Female	0.87 (0.81-0.93)	0.67 (0.57-0.77)	0.78 (0.69-0.88)	0.69 (0.59-0.82)	0.69 (0.56-0.79)
	Male	0.84 (0.75-0.93)	0.71 (0.62-0.82)	0.80 (0.75-0.85)	0.70 (0.66-0.74)	0.78 (0.72-0.85)
**Casual simulation video games**						
Racing	Total	0.75 (0.61-0.88)	0.72 (0.65-0.78)	0.71 (0.65-0.77)	0.69 (0.62-0.76)	0.79 (0.73-0.85)
	Female	0.61 (0.36-0.84)	0.63 (0.56-0.71)	0.65 (0.60-0.72)	0.62 (0.50-0.72)	0.74 (0.69-0.81)
	Male	0.81 (0.67-0.92)	0.75 (0.69-0.82)	0.74 (0.67-0.80)	0.72 (0.66-0.79)	0.81 (0.75-0.88)
Sports	Total	0.73 (0.59-0.86)	0.69 (0.62-0.76)	0.71 (0.66-0.75)	0.69 (0.61-0.77)	0.73 (0.69-0.77)
	Female	0.75 (0.52-0.92)	0.65 (0.53-0.76)	0.74 (0.70-0.79)	0.70 (0.57-0.85)	0.72 (0.68-0.76)
	Male	0.72 (0.56-0.85)	0.71 (0.65-0.79)	0.70 (0.65-0.75)	0.68 (0.60-0.76)	0.73 (0.68-0.78)
**Casual strategy video games**						
Tower defense	Total	0.82 (0.72-0.92)	0.68 (0.62-0.74)	0.78 (0.74-0.82)	0.76 (0.69-0.82)	0.74 (0.69-0.80)
	Female	0.87 (0.79-0.95)	0.65 (0.54-0.76)	0.76 (0.70-0.79)	0.74 (0.67-0.81)	0.76 (0.69-0.82)
	Male	0.80 (0.67-0.90)	0.69 (0.64-0.76)	0.79 (0.74-0.84)	0.76 (0.69-0.83)	0.74 (0.68-0.81)

### Effect Analyses for CEGE Scales

As hypothesized, there was a significant main effect for CVG genre on enjoyment (*F*_1, 2.977_=4.794, *P*=.005, *η* ²=.220); game-play (*F*_1,6_=4.698, *P*=.004, *η* ²=.227); control (*F*_1,6_=8.704, *P*=.001, *η* ²=.339); and ownership (*F*_1,6_=3.615, *P*=.003, *η* ²=.184) ratings. There was, however, no significant difference in environment ratings (*F*_1,6_=3.615, *P*=.07, *η* ²=.114) across CVG genres. To adjust the effects of video game genre for the potentially confounding effect of the graphics and audio of the video games (“environment”), the effect of video game genre and environment were analyzed within a LMEM for each CEGE scale. In addition, the effects of personal background variables (age, education, and prior game experience) were included in the LMEMs (Table 2). The LMEM analysis revealed a significant global effect of video game genre (*P*<.001) and a trend for positive effects of environment ratings (*P*=.05). For game-play ratings, there was a significant global effect of video game genre (*P*<.001) and a significant negative effect of game experience (*P*=.01). For control ratings, there was only a significant global effect of video game genre (*P*<.001), whereas there was a significant global effect of video game genre (*P*=.01) and a significant positive effect of environment ratings (*P*=.03) on ownership ratings. The linear mixed-effects model was further used to perform all 21 pairwise comparisons between the 7 CVGs for each CEGE scale showing significant global effects of the factor video game genre. Pairwise comparison of the estimated marginal means for enjoyment indicate that the casual puzzle video game (Flow Free) was significantly more enjoyed than casual simulation racing (*t*_89_=−3.74, *P*=.01) and sports (*t*_89_=−3.61, *P*=.01) video game. Game-play ratings of casual action no shooting video game scored significantly lower than those for the casual puzzle video game (Bejeweled; *t*_89_=3.47, *P*=.02), and the casual simulation racing (*t*_89_=3.74, *P*=.01) and sports (*t*_89_=3.32, *P*=.03) video game. Pairwise comparisons further indicated that control ratings for the casual puzzle video game (Flow Free) were significantly better than those for both the casual action no shooting (*t*_89_=4.58, *P*<.001) and shooting (*t*_89_=4.68, *P*<.001) video game as well as for the casual simulation racing (*t*_89_=−5.39, *P*<.001) and sports (*t*_89_=−5.02, *P*<.001) video game. Furthermore, the second casual puzzle video game (Bejeweled) was rated better in terms of control than the casual simulation racing video game (*t*_89_=−3.28, *P*=.03). Finally, pairwise comparisons on the effect of video game genre on ownership revealed that both the casual puzzle video game (Flow Free; *t*_89_=3.46, *P*=.02) and casual strategy video game (Plants vs Zombies; *t*_89_=3.39, *P*=.02) were rated better than the casual action no shooting video game.

**Table 2:** Tests for fixed effects within the linear mixed effects models (LMEM) for each CEGE scale.

Variable	Enjoyment	Game-play	Control	Ownership
Video game genre	*F*_6, 89_=3.54^a^	*F*_6, 89_=3.89^a^	*F*_6, 89_=8.03^b^	*F*_6, 89_=3.24^a^
Environment rating	*t*_89_=1.96	*t*_89_=1.73	*t*_89_=0.94	*t*_89_=2.22^a^
Game experience	*t*_12_=1.94	*t*_12_=−3.23^a^	*t*_12_=−1.86	*t*_12_=0.13
Age	*t*_12_=−1.06	*t*_12_=0.66	*t*_12_=0.73	*t*_12_=0.56
Education	*t*_12_=−0.26	*t*_12_=0.32	*t*_12_=−0.87	*t*_12_=0.24

^a^Significant at the .001 level.

^b^Significant at the .05 level (two-sided).

## Discussion

### Principal Findings

In this study, we examined casual game enjoyment and game characteristics ratings across a range of 4 genres in healthy older adults. The results of the playtest study revealed that tablet-based casual games are generally enjoyed by older adults with an effect of CVG genre on enjoyment, independent of environment ratings and personal background variables. In addition, the constituent elements of casual games resonated well with healthy older adults. There was an independent effect of CVG genre on gameplay, control, and ownership ratings. Moreover, environment ratings had an effect on enjoyment and ownership, but not on control and gameplay, when controlling for all other fixed effects. Prior game experience positively influenced enjoyment and negatively influenced game-play ratings. Finally, there were no independent effects of age and education on any of the CEGE scales.

### Comparison With Prior Work

The genre effect on enjoyment indicates a preference for the casual puzzle (Flow Free) over the casual simulation games (Real Racing and Virtual Table Tennis). Nevertheless, all CVGs were given good enjoyment and replayability ratings. This finding is strongly in line with findings from previous survey studies in active older gamers [16,24,37] and 2 playtest studies using similar puzzle games [15], reporting that casual games are generally the most liked type of video games among older adults. This further reflects the preference of older adults for slower-paced games with an intellectual challenge over fast-paced games (eg, action, sports, and strategy games) and relates to the notion that older adults prefer to play games that are similar to the games they used to play when they were younger (eg, card, board, and paper-based puzzle games) [15].

However, we did not find evidence to support a general dislike for the action game genre (no shooting and shooting) reported in other studies [7], as this represents the second most liked casual game genre in our study. It has been argued that the strong visual, attentional, and processing speed demands, which are known to decline with age, make action games less enjoyable for older players [38]. Although the casual action games in this study relied heavily on fast reaction and hand-eye coordination, it is our belief that their nonviolent and joyful themes contributed to an enjoyable experience in our study. This again, is in line with the findings of McKay and Maki [7], showing that older adults did enjoy and were willing to play a cartoonish FPS game, whereas they disliked and were unwilling to play a realistic FPS game with violent content. To sum up, the higher ratings for puzzle than action games replicate the findings of a recent playtest study [15] comparing an action with a puzzle game, that were similar in terms of the cognitive abilities they engage, and found that the puzzle game was deemed more motivating by older adults.

As for the third most liked casual strategy game genre (Plants vs Zombies), we were not able to relate this finding to survey and other playtest study findings; as, to the best of our knowledge, there have been no studies on enjoyment and usability using strategy games in older adults. However, strategy games have successfully been used in 1 video game training study with older adults [39]. Finally, we found that casual simulation (sports and racing) games were least liked by the participants in this study. This again is reflected in survey findings reporting that even among active older game players, only 20% reported playing racing and sports games [16].

Taken together, these findings indicate a positive overall attitude of older adults toward tablet-based casual games, and that they are very open and willing to try out new technology when given an opportunity to play. Of note, environment (ie, graphics and sounds) ratings remained a contributor to enjoyment when controlling for all other effects. Whereas environment ratings did not differ across genres, the visual presentation of a CVG seems to contribute to overall enjoyment of CVGs.

We also find hands-on evidence to support the notion that casual games provide a set of game characteristics that are suitable for a senior audience in terms of usability [34]. Overall, the casual game characteristics were well-perceived by the older adult players in terms game-play, control, and ownership.

The gameplay ratings, that is, how participants judged the rules and underlying story (scenario) of the games were satisfactory, with the casual simulation and puzzle video games being easier to understand than the casual action (no shooting game) video game. The fact that the least liked games were rated easiest to understand is hardly surprising, as simulation games make use of real-world concepts and rules already known to the player and thus, at least in terms of rules of the game, are particularly suitable for an older audience with little or no game experience [34]. In terms of control ratings, that is, whether the goal and actions of the game were clear and the controllers of the game were easy to use, the casual puzzle video game (Flow Free) was rated better than all casual simulation and action video games. Finally, regarding ownership ratings, that is the extent to which player sees the actions in the game as a result of his own efforts, the casual puzzle (Flow Free) and the casual strategy video games (Plants vs Zombies) were rated better than the casual action no shooting game (Pocket Frog Splash).

The latter two findings reflect the above-mentioned notion that action games are not as much appreciated due to the speed of movement and intense interaction, which is reflected in the lower control and ownership ratings [15]. These findings underscore previous reports that puzzle games are the preferred game genre of older adults and the easiest to interact with [40].

Of note, the least enjoyed casual simulation (sports and racing) video games differed inherently from the others in how players had to control the game. Other than using single tapping or sliding movements as game input, these games were unique in that they required players to perform tilting (to steer the car in the racing game) or continuous drag movements (to move the ping-pong paddle in the sports game). This additional load on the interaction with the game should be considered when comparing ratings of enjoyment and interaction with the game.

### Limitations

This study is not without known methodological limitations regarding problems in obtaining a representative sample of older adult players [6]. Of note, there is great heterogeneity in older people regarding their cognitive and physical abilities and their preferred leisure activities that were not addressed in this study. Although our participants were healthy, highly motivated older adults interested in research with little or no prior game experience and were generally unfamiliar with video game genre jargon, it remains unclear whether our findings can be generalized to less-motivated and even cognitively impaired persons.

Although special attention was paid to gender-inclusivity during the selection of the casual games, we did not include gender as a factor in the analysis of genre preference and interaction with the game, owing to the smaller number of female participants.

In addition, playing time for each video game was limited to around 5 min, allowing us to collect first impressions and ease of interaction, rather than long-term experiences with the video games. It therefore remains unclear whether the CVG genre preferences persist over extended experience with the game (eg, during a game-based intervention).

### Conclusions

The goal of this study was to find out how well different CVG genres met casual game characteristics and how this led to game enjoyment in a population of older adults. We argue that our findings can be matched with a recent position De Schutter and Abeele [9] take in their “gerontoludic” manifesto. Here, the authors criticize that video game-based aging interventions focus too much on improving cognitive function and usability, while fun aspects are often forgotten.

To address this issue, the manifesto states that video games should primarily provide older adults with a meaningful and playful activity. We were able to confirm this with the general finding that casual games were well-enjoyed and participants were willing to play them. This is closely related to their second claim that video games should focus on challenge and personal growth and rather than simply combatting age-related cognitive decline. Although we did not address this, we believe that this claim is easily met by the positive themes CVGs are characterized by and that these games use difficulty adjustments to optimally challenge the player. Finally, in their third claim the authors propose that video games should offer a diversity to accommodate the heterogeneity of older adults in their cognitive and physical skill levels, backgrounds, and preferences. Again, it is our understanding that it is in the very nature of CVGs to satisfy this demand.

In line with recent findings, this study confirms a special preference of older adults for the puzzle game genre. It would be interesting for future studies to capitalize on this in future video game interventions for older adults. It also appears worthwhile to conduct video game interventions using multiple game genres, as only few studies have looked at the training benefits of different game genres in older adults [30]. We therefore welcome future studies to continue exploring the potential of CVG interventions and investigate possible effects on cognition, everyday functioning, and well-being. Finally, we see particular clinical potential for CVGs in people suffering from cognitive impairment due to dementive and depressive disorders or brain injury.

## Abbreviations

ANOVA: analysis of variance
CEGEQ: Core Elements of the Gaming Experience Questionnaire
CVG: casual video game
FPS: first person shooter
LMEM: linear mixed effects model
